# Structural insights into the stimulation of *S. pombe* Dnmt2 catalytic efficiency by the tRNA nucleoside queuosine

**DOI:** 10.1038/s41598-018-27118-5

**Published:** 2018-06-11

**Authors:** Sven Johannsson, Piotr Neumann, Alexander Wulf, Luisa M. Welp, Hans-Dieter Gerber, Matthias Krull, Ulf Diederichsen, Henning Urlaub, Ralf Ficner

**Affiliations:** 1 Department of Molecular Structural Biology, Institute of Microbiology and Genetics, GZMB, Georg-August-University Göttingen, 37077 Göttingen, Germany; 2 Bioanalytical Mass Spectrometry Research Group, Max Planck Institute for Biophysical Chemistry, Am Faßberg 11, 37077 Göttingen, Germany; 3 Institut für Pharmazeutische Chemie der Philipps-Universität Marburg, Marbacher Weg 6, 35032 Marburg, Germany; 4 Institut für Organische und Biomolekulare Chemie, Georg-August-Universität Göttingen, Tammannstrasse 2, 37077 Göttingen, Germany; 5 Bioanalytics, Department of Clinical Chemistry, University Medical Center Göttingen, Robert-Koch-Straße 40, 37075 Göttingen, Germany

## Abstract

Dnmt2 methylates cytosine at position 38 of tRNA^Asp^ in a variety of eukaryotic organisms. A correlation between the presence of the hypermodified nucleoside queuosine (Q) at position 34 of tRNA^Asp^ and the Dnmt2 dependent C38 methylation was recently found *in vivo* for *S. pombe* and *D. discoideum*. We demonstrate a direct effect of the Q-modification on the methyltransferase catalytic efficiency *in vitro*, as V_max_/K_0.5_ of purified *S. pombe* Dnmt2 shows an increase for *in vitro* transcribed tRNA^Asp^ containing Q34 to 6.27 ∗ 10^–3^ s^−1^ µM^−1^ compared to 1.51 ∗ 10^–3^ s^−1^ µM^−1^ for the unmodified substrate. Q34tRNA^Asp^ exhibits an only slightly increased affinity for Dnmt2 in comparison to unmodified G34tRNA. In order to get insight into the structural basis for the Q-dependency, the crystal structure of *S. pombe* Dnmt2 was determined at 1.7 Å resolution. It closely resembles the known structures of human and *E. histolytica* Dnmt2, and contains the entire active site loop. The interaction with tRNA was analyzed by means of mass-spectrometry using UV cross-linked Dnmt2-tRNA complex. These cross-link data and computational docking of Dnmt2 and tRNA^Asp^ reveal Q34 positioned adjacent to the S-adenosylmethionine occupying the active site, suggesting that the observed increase of Dnmt2 catalytic efficiency by queuine originates from optimal positioning of the substrate molecules and residues relevant for methyl transfer.

## Introduction

An amazing diversity of chemically modified nucleosides has been identified in various RNAs in all kingdoms of life^1,2^. At least 93 different modifications were found in tRNA molecules. Interestingly, modification of tRNA is not just a static posttranscriptional step in tRNA maturation, but can also be dynamic and might be part of a response to stimuli such as stress^3^. Modifications in the anticodon-loop of tRNAs are known or proposed to affect the codon-anticodon interaction and therefore the fidelity and rate of translation, while modifications in other regions of the tRNA mainly modulate the stability and flexibility of tRNAs^4,5^. However, modifications of tRNAs might have also other important functions. For example, C5-methylation of C38 in the tRNA anticodon-loop by the methyltransferase Dnmt2 was shown to protect tRNAs from cleavage^6^, which in turn has an impact on Dicer-2-dependent siRNA pathways^7^. Methylation of C38 also promotes the aminoacylation of tRNA^Asp^ *in vitro*^8^, and contributes to translational accuracy^9^.

Recently, the C38 methylation of tRNA^Asp^ in *S. pombe* was found to depend on the presence of queuosine (Q), a hypermodified nucleoside at position 34 of tRNA^Asp^, tRNA^Asn^, tRNA^Tyr^, and tRNA^His^^10^. Even though Q was discovered more than 40 years ago^11^, its common physiological role in eubacteria and eukaryotes is still unclear^12^. Due to its position in the anticodon as wobble base, Q has been believed to modulate codon-anticodon interaction, however, a clear effect on mRNA translation has yet not been demonstrated. Crystal structures of a ribosome with a C-A mismatch of Q34-tRNA^Tyr^ bound to a His codon revealed a displacement of the cytosine from the codon–anticodon helix and distortion of the latter, hinting to a role of the Q-modification in translational accuracy^13^. While prokaryotes are able to synthesize Q *de novo*, it is a nutrient for eukaryotes. Importantly, the enzyme tRNA-guanine-transglycosylase (TGT) replaces the guanine at position 34 of the tRNA by a base exchange mechanism^14^. In case of prokaryotes the G34 is replaced by the queuine precursor preQ1, and in eukaryotes by queuine. In contrast to bacterial TGT, which is a homo-dimer, human TGT is a functional hetero-dimer consisting of a catalytically active subunit (QTRT1), and a homologous, but catalytically inactive subunit (QTRTD1)^15^.

The Q-dependency of tRNA^Asp^ C38 methylation was found *in vivo*, and the effect holds true for purified DNMT2 and tRNA extracted from *S. pombe* grown under queuine-deficient conditions^10^. However, this did not exclude the possibility that other tRNA modifications might be involved in the stimulation of DNMT2 as well, as the tRNA used was lacking Q, but was otherwise fully modified.

Here we demonstrate that Q34 of tRNA^Asp^ solely is sufficient to increase Dnmt2 catalytic efficiency. We present the crystal structure of *S. pombe* Dnmt2 and propose a model of the Dnmt2-tRNA complex, based on *in silico* docking and mass spectrometric analysis of cross-links obtained from the Dnmt2-tRNA complex, showing that Q34 is part of the active site.

## Results

### Methlytransferase efficiency is stimulated by Q34 in tRNA^Asp^

**Figure 1:**
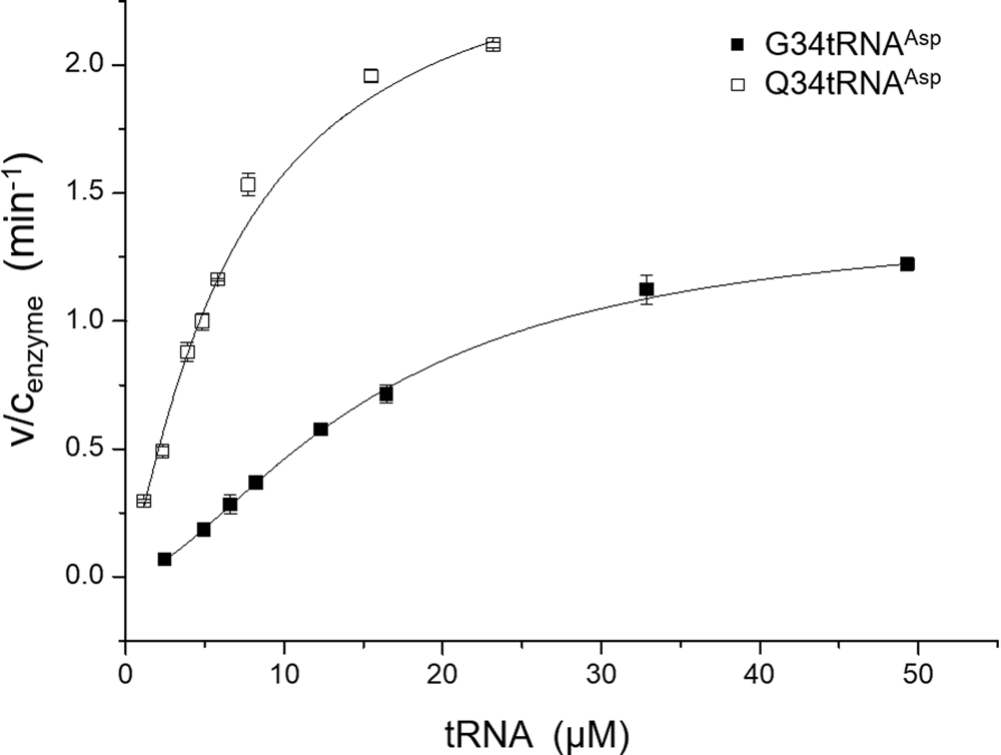
Methyltransferase activity assay with spDnmt2 using unmodified tRNA^Asp^ (G34tRNA^Asp^) and queuine harboring tRNA^Asp^ (Q34tRNA^Asp^) as substrates. Activity is plotted as substrate conversion per enzyme concentration in min^−1^. Measurements were performed as independent triplicates with increasing substrate concentrations. Errors are presented as standard deviation. Data points were fitted with the Hill equation employing a free Hill coefficient. V_max_ increased from 1.41 ± 0.01 min^−1^ for G34tRNA^Asp^ to 2.59 ± 0.11 min^−1^ for Q34tRNA^Asp^. K_0.5_ shifted from 15.59 ± 0.29 µM to 6.88 ± 0.58 µM respectively.

Previous data from Müller *et al*. with purified recombinant Dnmt2 and tRNA extracted from a Dnmt2 deletion mutant of *S. pombe* showed decreased activity of Dnmt2 with tRNA prepared from *S. pombe* grown under queuine deficient conditions. To rule out an involvement of other modifications present on the used tRNA^Asp^ we established a system completely outside of *S. pombe*. Dnmt2 of *S. pombe* (spDnmt2) was recombinantly expressed and purified while tRNA was generated by *in vitro* transcription. As expected spDnmt2 was active on this unmodified substrate. To quantify the effect of Q modification in context of tRNA^Asp^ queuine was enzymatically introduced into tRNA^Asp^ (Q34tRNA^Asp^) using the recombinant human tRNA guanine transglycosylase (TGT). Efficiency of spDnmt2 on Q34tRNA^Asp^ was stimulated compared to unmodified G34tRNA^Asp^ (Fig. 1). We observed an increased catalytic efficiency (k_cat_/K_0.5_) from 1.51 ∗ 10^−3^ s^−1^ µM^−1^ up to 6.27 ∗ 10^−3^ s^−1^ µM^−1^ for tRNA^Asp^ when G34 is exchanged by Q34. K_0.5_ shifted from 15.60 ± 0.29 µM for G34tRNA^Asp^ down to 6.88 ± 0.58 µM for Q34tRNA^Asp^. These results suggest the increased efficiency to be directly triggered by Q34 modification. We also observed that fit with the Michealis-Menten equation only poorly described the observed data (Supplementary Fig. S6). It was best described by employing the Hill equation as a free fit (Fig. 1). We observed a Hill coefficient of n = 1.63 ± 0.02 for the unmodified and n = 1.19 ± 0.07 for the Q34 modified tRNA substrate.

### Affinity of tRNA^Asp^ to Dnmt2

**Figure 2:**
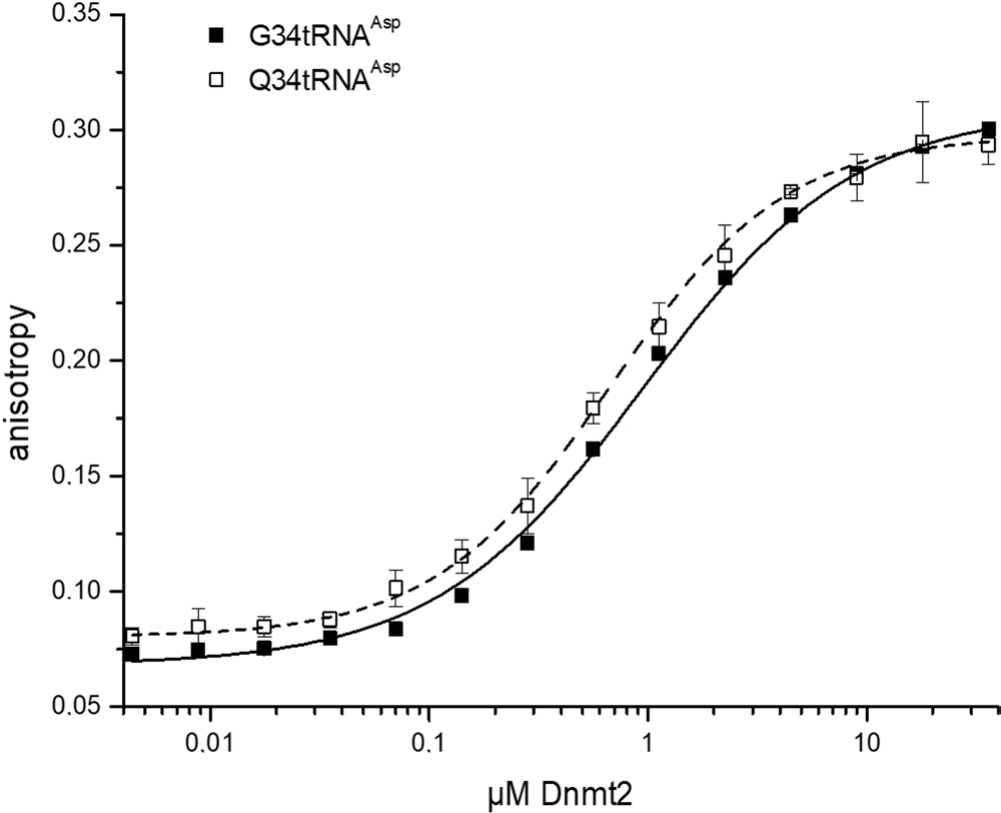
Quantitative analysis of Dnmt2 tRNA complex formation. Binding of the indicated fluorescein labelled G34tRNA^Asp^ and Q34tRNA^Asp^ was analyzed with increasing spDnmt2 concentration. Complex formation was observed with fluorescence polarization.

We further ought to understand how Q34 alters Dnmt2 catalytic efficiency on tRNA^Asp^. Quantitative analysis of binding with fluorescently labelled tRNAs revealed a change in affinity by Q34 modification (Fig. 2). Presence of queuine in the tRNA resulted in a decrease in K_d_ from 0.96 ± 0.05 µM to 0.67 ± 0.003 µM, which, however, may not fully explain the positive effect of Q34 on Dnmt2 efficiency observed in methyltransferase activity assay.

### Structural insights into *S. pombe* Dnmt2

**Figure 3:**
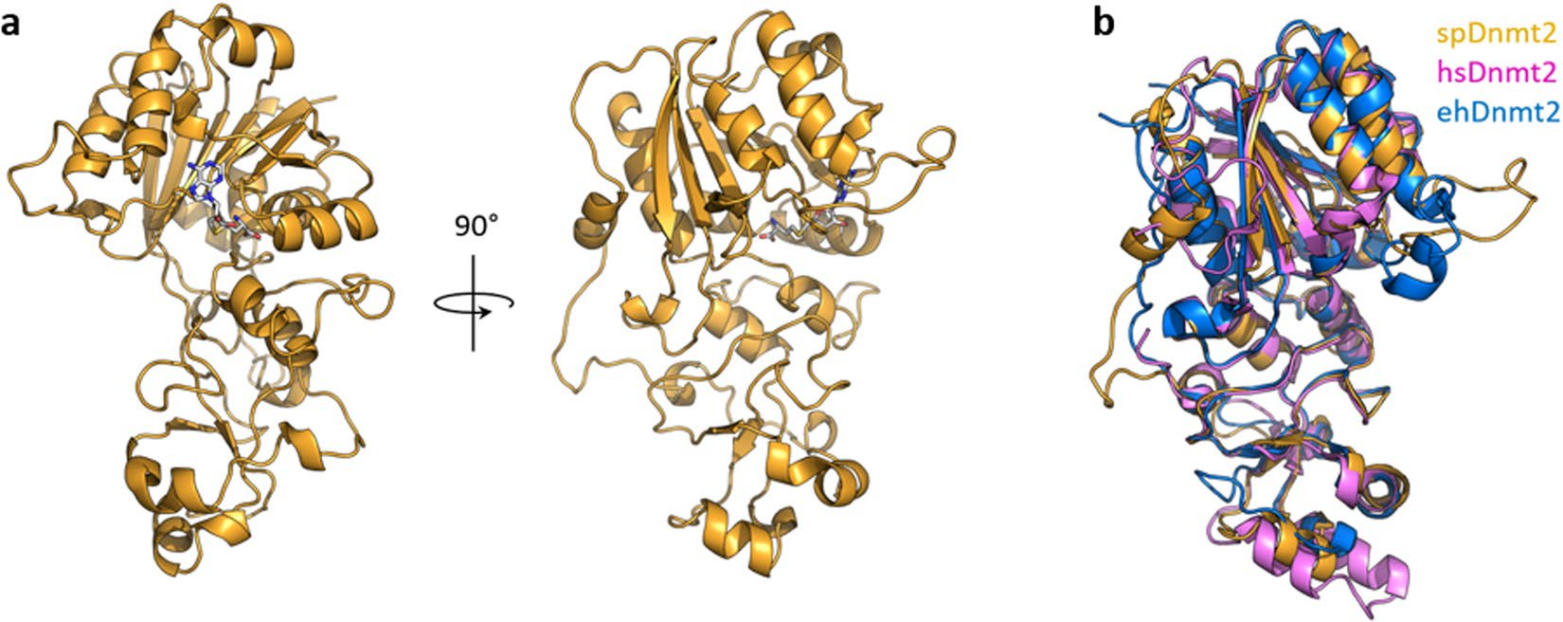
(**a**) Cartoon representation of *S. pombe* Dnmt2 crystal structure (yellow) co-crystallized with S-adenosyl-homocysteine depicted as sticks (grey). The crystal structure was refined at 1.7 Å. (**b**) Superposition of Dnmt2 crystal structures from *S. pombe* (yellow), *H. sapiens* (pink) and *E. histolytica* (blue) shown as cartoons.

For further insights into the activation of Dnmt2 by Q we solved the spDnmt2 crystal structure in complex with S-adenosyl-homocysteine (SAH) at 1.7 Å resolution by molecular replacement (Fig. 3a). This structure comprises all amino acids from position 5 to the C-terminal end, including loop regions, and exhibits an overall simiIar fold with the Dnmt2 crystal structures from human (hsDnmt2)^16^ and the pathogenic amoebae *E. histolytica* (ehDnmt2)^17^ (Fig. 3b). In the spDnmt2 structure we were able to build a loop of 20 amino acids starting with Cys80, which was proved to be important for catalysis in hsDnmt2 and therefore is referred to this loop as the “active site loop”. This loop is presumably unstructured in solution as the electron density for the corresponding amino acids was only observed for one of the four Dnmt2 molecules in the asymmetric unit, and this loop adopted a defined conformation due to formation of crystal contacts with another Dnmt2 molecule in the crystal lattice. A corresponding loop is also resolved in ehDnmt2 structure where it exhibits a different conformation which appears to be confined by crystal contacts^17^. The crystal structure of hsDnmt2 does not exhibit any traceable loop comprising the corresponding amino acids, arguing flexibility of the active site loop in Dnmt2 of all three organisms.

Analysis of spDnmt2 electrostatic surface potentials unveils predominantly positively charged surface around the SAH indicating possible interaction with the tRNA phosphate backbone. However, the structure exposes a strongly negatively charged cavity in close proximity to the sulfur of SAH formed by the catalytic residue Glu121, which is also conserved in hsDnmt2 and ehDnmt2. This cavity might accommodate the base of cytosine C38, which needs to be flipped out in order to be methylated. The required base flipping has been structurally observed for Dnmt1, which uses the same catalytic mechanism^18^.

### Analysis of the DNMT2-tRNA^Asp^ complex with UV induced cross-linking

**Figure 4:**
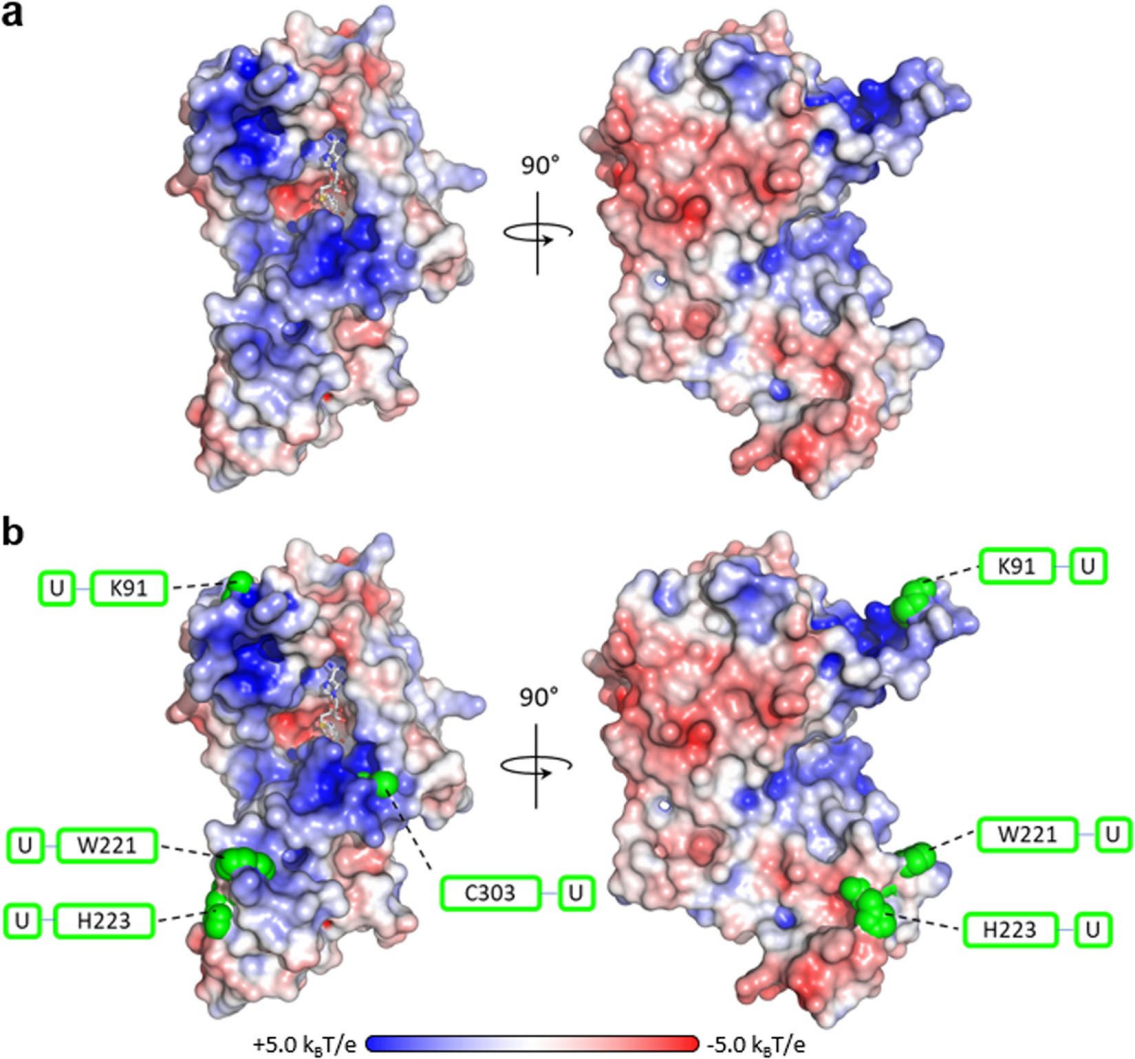
(**a**) Surface representation of *S. pombe* Dnmt2 electrostatic potentials depicted at a contour level of ±5.0 k_B_T/e. SAH is presented as grey sticks. (**b**) Structural analysis of protein RNA cross-links. Residues cross-linked to tRNA^Asp^ are depicted as green spheres. All cross-links were observed as covalent links to uridines.

To elucidate how queuine impacts Dnmt2 catalytic efficiency we aimed at understanding how Dnmt2 binds its tRNA substrate. Therefore, we performed UV light induced cross-linking of the Dnmt2 tRNA^Asp^ complex and analyzed the cross-linked peptides with mass spectrometry. Four residues cross-linked to the RNA were identified, namely Lys91, Trp221, His223, and Cys303, with each of them cross-linked to a uridine (Fig. 4).

**Figure 5:**
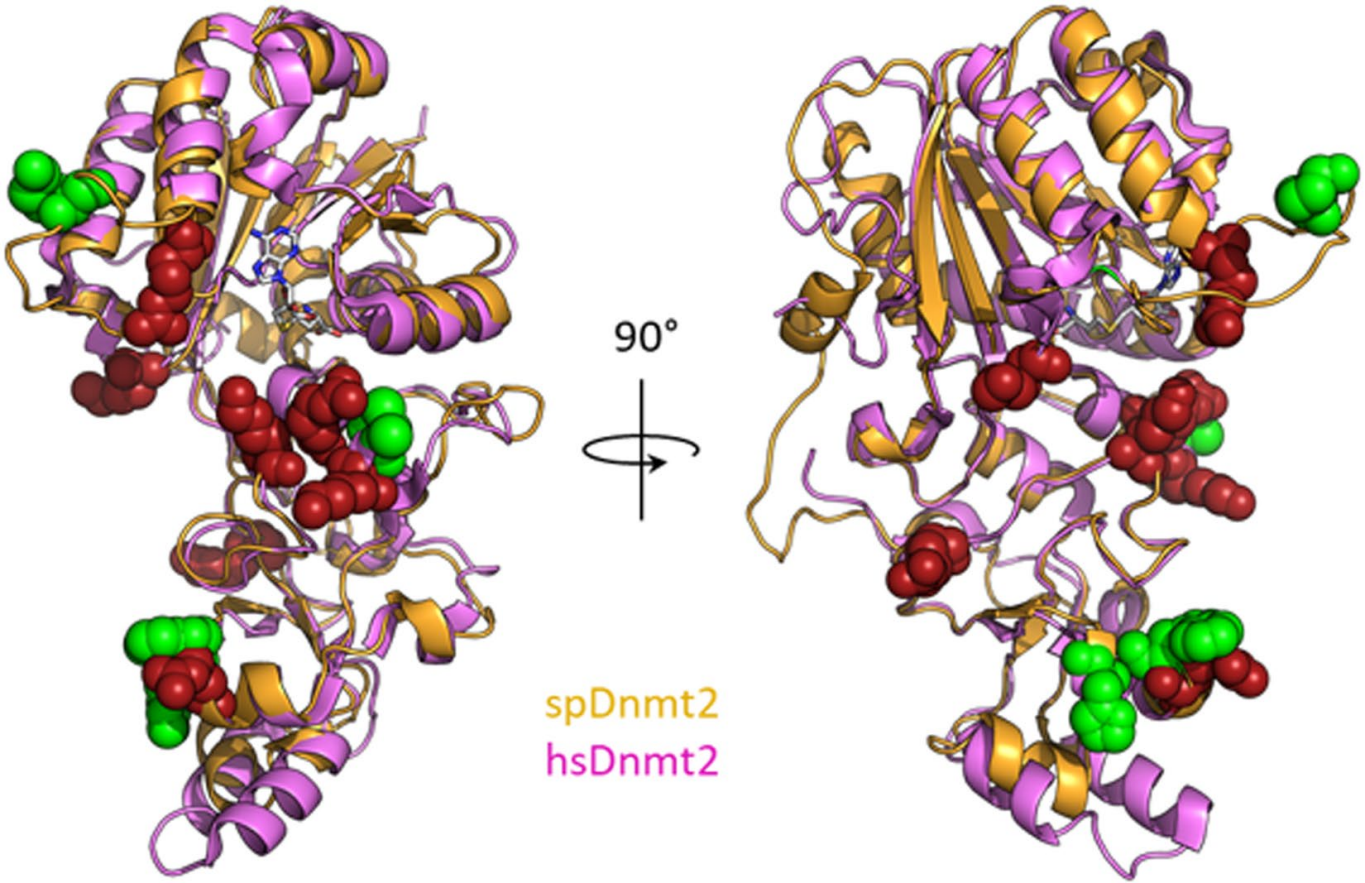
Overlay of spDnmt2 cross-links to tRNA^Asp^ with lysine and arginine residues known to be important for Dnmt2 catalytic activity in human. spDnmt2 and hsDnmt2 were superimposed and depicted as cartoon. spDnmt2 residues cross-linked to tRNA^Asp^ are depicted as green spheres, hsDnmt2 residues for which single mutation to alanine resulted in <25% activity compared to wildtype are shown as red spheres.

An overlay of spDnmt2 and hsDnmt2 reveals that three of these cross-links are positioned in close proximity to residues important for activity in hsDnmt2 (Fig. 5), as mutation of a single of these residues resulted in less than 25% activity compared to wildtype^19^. The Cys303 cross-link is in close proximity to a positive patch formed by Arg371, Lys295 and Lys367 in hsDnmt2. Additionally, we find cross-linked residues Trp221 and His223 to be close to hsDnmt2 Arg275 residue. Lys91 is positioned within the active loop and in close proximity to Arg95, a residue conserved in both *S. pombe* and human but not resolved in the structure of hsDnmt2. Strikingly, mutation of this residue in human Dnmt2 mostly abolishes activity, highlighting the importance of the flexible loop for Dnmt2 catalytic function.

### Dnmt2-tRNA docking places G34 in close proximity to the active site

For a more detailed understanding of how Dnmt2 binds to tRNA and which regions might be involved in Q recognition we performed computational docking of the Dnmt2 tRNA complex. Cross-linking data was used for model validation but docking was done without applying restrains for the cross-links. For docking we chose the herein described spDnmt2 crystal structure and tRNA structures from the PDB. The best Dnmt2-tRNA model was derived from docking of the crystal structure of yeast tRNA^Asp^ (1VTQ). Afterwards the best docked tRNA^Asp^ model was manually modified, the G34 was replaced by queuine and the C38 base was flipped, as the flipped out conformation of this base has shown to be necessary for methylation by Dnmt proteins^18^.

**Figure 6:**
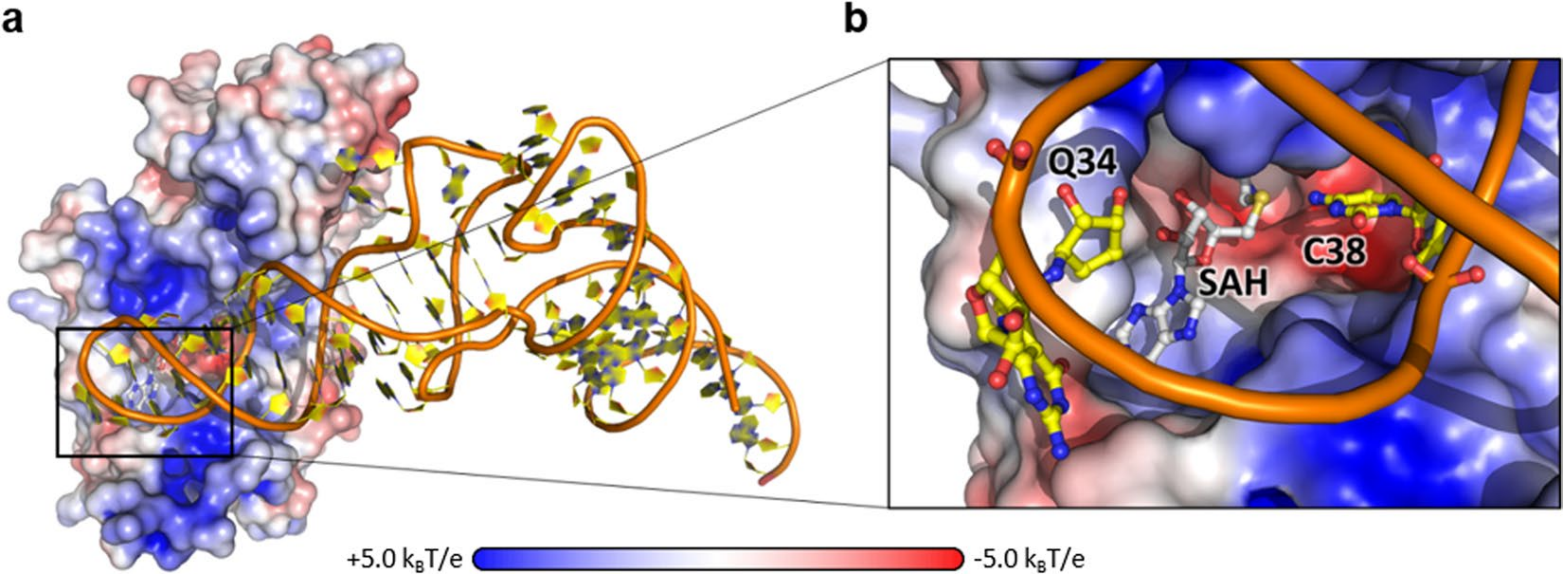
(**a**) Predicted model of the Dnmt2-tRNA complex. spDnmt2 and yeast tRNA^Asp^ (1VTQ) were docked computationally using Rosetta. spDnmt2 surface is depicted with surface electrostatics (±5.0 k_B_T/e). tRNA^Asp^ is shown as cartoon with orange ribose-phosphate backbone and yellow bases. (**b**) Zoomed view into the active site. Queuine 34, cytosine 38 and S-adenosyl-homocysteine (SAH) are presented as balls and sticks.

The resulting model of the tRNA Dnmt2 complex (Fig. 6) has the tRNA placed with the anticodon stem in a positively charged groove around the active site. In this orientation tRNA phosphate backbone forms numerous electrostatic interactions with Dnmt2. Interestingly, in this model Q34 is facing the protein surface close to active site with the diol ring close to the SAM binding grove. Furthermore, the cross-linked residues Cys303, Trp221 and His223 are adjacent to bases that correspond to uridines in sptRNA^Asp^ that was used in cross-linking. However cross-linked Lys91 is not positioned close to an uridine in our model, which indicates that the conformation of the active site loop might not reflect the arrangement of the loop in the Dnmt2-tRNA complex in solution. Given the loop’s likely flexibility that we described earlier the loop might exhibit an alternative conformation and be positioned closer to the tRNA when the substrate is bound.

## Discussion

We proved that queuine alone is able to increase spDnmt2 catalytic efficiency when incorporated into position 34 of tRNA^Asp^ and did quantify this effect with a newly established system completely outside of *S. pombe*. We also observed that the spDnmt2 reaction kinetics do not follow classic Michaelis-Menten kinetics but are better described with a cooperative fit using the Hill equation. From published data and our experience there is no evidence that Dnmt2 is active as a multimer. However, kinetic cooperativity has also been confirmed for monomeric proteins with a single active site. In these cases this observed kinetic cooperativity can be caused by a slow interconversion of two or more conformations of the enzyme upon formation of the enzyme substrate complex, which shifts the equilibrium of these towards the catalytically active conformation^20^. We further could show that Q34 increases reaction velocity and lowers K_0.5_ by almost three fold. At the same time the modification does lower binding affinity by 1.4 fold. These two observations raise the question of how this modification results in the increase of Dnmt2 catalytic efficiency.

In order to shed light on this question, we solved the reported structure of *S. pombe* Dnmt2. This offers direct insights into the spDnmt2 structural properties and finds its fold to be closely related to Dnmt2 molecules of other organisms. The structure includes a flexible loop adjacent to the active site, which has been found to be crucial for activity of Dnmt2 in human^19^.

Interestingly Pro83 in this loop exhibits a cis-peptide conformation in chains A and B of the crystal structure. This residue is conserved in Dnmt2, but neither in Dnmt1 nor in Dnmt3^21^. Proline has been shown to be the only natural amino acid to form such a stable *cis* peptide backbone and conversion between cis and trans is hindered by a high energy barrier^22,23^. Rate constants for proline isomerization therefore are minimal and have been shown be as slow as 0.002 s^−1^ ^24^. To our knowledge kinetic cooperativity in a monomeric enzyme caused by proline isomerization has not been shown so far, but earlier articles already highlight proline cis-trans isomerization to harbor this potential^20^. Therefore, the isomerization of Pro83 could serve as a possible explanation for the observed cooperative effect.

Our experimental approach with UV light induced protein RNA cross-linking combined with mass spectrometry provides to our knowledge the first structural data on Dnmt2 tRNA complex formation. Comparison of protein residues involved in these cross-links with a mutational study on human Dnmt2^19^ revealed that the observed cross-links appear in the same or adjacent regions as residues that could be directly linked to hsDnmt2 activity. Additionally, comparison of the spDnmt2 crystal structure with structures of Dnmt2 from other organisms available in the PDB revealed that all structures exhibit an overall similar conformation, arguing in favor of a comprehensively similar substrate binding mechanism by Dnmt2 across species.

The modeled Dnmt2-tRNA complex reveals Q34 positioned close to the active site but simultaneously too distant from the catalytic residues to directly participate in the methyltransfer reaction. This seems to be likely as Dnmt2 is also active on unmodified G34tRNA^Asp^.

As revealed by *in silico* docking experiments, the tRNA’s variable loop region and the L-loop, both of which have been shown to be important for methylation by human Dnmt2^25^, are located in vicinity of Dnmt2. Additionally, our docking model of the Dnmt2-tRNA complex is in a good agreement with three of the observed four crosslinks. However, the model does not support the fourth (Lys91) cross-link. This residue is harbored inside the active site loop, which in our spDnmt2 structure is stabilized by crystal contacts. The docking reveals that this conformation does not interfere with the predicted potential binding of the tRNA substrate, however, our result suggests it likely adopts an alternative conformation closer to the RNA when the substrate is bound. This is supported by the mentioned previous mutational analysis of hsDnmt2^19^ which reveals that the active loop harbored and conserved Lys95 is crucial for Dnmt2 activity and as thus is likely to show interaction with the substrate. Furthermore, our Dnmt2-tRNA complex docking model shows a very similar arrangement of nucleotides close to the active site compared with the crystal structure of the Dnmt1-DNA complex which uses the same catalytic mechanism^18^.

Finally, the question arises whether the described effect by queuine is restricted to *S. pombe*. Published data describing the analysis of tRNA^Asp^ modifications in human revealed C38 methylation only to be present in combination with mannosylated queuine at position 34^25^. This finding as well as the observed structural similarities of Dnmt2 across organisms in addition with the observation that tRNA binding of Dnmt2 in *S. pombe* and human are likely to be similar does argue that the influence of Q34 or hyper-modified Q34 on Dnmt2 might be a more general mechanism in other organisms.

## Materials and Methods

### Expression and Purification

*Saccharomyces pombe* Dnmt2 (spDnmt2) was expressed from pGEX 6P-3 vector as GST-spDnmt2 fusion protein in *Escherichia coli* BL21(DE3) cells using autoinduction. Cells were grown at 16 °C for 50 h before harvesting and stored at −80 °C until further use. Cells were disrupted by microfluidization (M-110S Microfluidizer) with 50 mM Tris/HCl pH 7.5, 150 mM NaCl, 1 M LiCl, 2 mM DTT and soluble protein was isolated by ultracentrifugation at 50 000 g for 30 min. GST-spDnmt2 was loaded onto Glutathione Sepharose FastFlow (GE Healthcare) (50 mM Tris/HCl pH7.5, 150 mM NaCl, 2 mM DTT) and eluted using 30 mM reduced glutathione. GST-tag was cleaved upon addition of PreScission Protease (1:80 w/w, GE Healthcare). GST was removed by heparin-sepharose (GE Healthcare) purification (50 mM Tris/HCl pH7.5, 100 mM-2M NaCl, 2 mM DTT). spDnmt2 was further purified by Superdex S75 size exclusion chromatography (50 mM Tris/HCl, 150 mM NaCl, 2 mM DTT). Purified spDnmt2 was concentrated to 3.5 mg/mL and stored at −80 °C until further use.

Active human tRNA guanine transglycosylase (hTGT) is a heterodimer consisting of QTRT1 and QTRTD1^15^. QTRT1 and QTRTD1 were co-expressed from pCDF-Duett vector with N-terminally 6xHis tagged QTRT1 (his-hTGT) in *Escherichia coli* BL21(DE3)-STAR cells using autoinduction. Medium was supplemented with additional 100 µM ZnCl_2_. Cells were grown at 18 °C for 50 h before harvesting and stored at −80 °C until further use. Cells were disrupted by microfluidization (M-110S Microfluidizer) (50 mM HEPES pH7.5, 100 mM NaCl, 10 mM Imidazole) and soluble protein was isolated by ultracentrifugation at 50 000 g for 30 min. His-hTGT was loaded onto His-Talon Superflow (GE Healthcare) and washed with additional 1 M LiCl. Target protein was eluted in a 20 CV gradient to 50 mM HEPES pH7.5, 100 mM NaCl, 500 mM Imidazole. Protein was further purified by Superdex S200 (GE Healthcare) size exclusion chromatography (20 mM HEPES pH 7.5, 100 mM NaCl). His-hTGT was concentrated to 16–20 mg/mL and stored at −80 °C until further use.

### *In vitro* transcription and tRNA purification

*Saccharomyces pombe* tRNA^Asp^ (sptRNA^Asp^) was transcribed from linearized vector template in run off *in vitro* transcription. Reaction contained T7 Polymerase and 10 mM rNTPs each in 1× HT buffer (30 mM HEPES pH 8.0, 25 mM MgCl_2_, 10 mM DTT, 2 mM Spermidine, 0.01% Triton X-100). After incubation for 3–16 h at 37 °C reaction was stopped with 50 mM EDTA final, concentrated and run in a 10% Urea-Polyacrylamid gel. tRNA was cut from the gel, extracted (10 mM Tris/HCl pH8.0 1 mM EDTA, 300 mM NaCl) and subjected to ethanol precipitation. Purified tRNA was dissolved in H_2_O and stored at −20 °C until further use.

### Q-Base synthesis

The queuine base was synthesized either as described previously^26^, or with a modified synthesis strategy for the (1R,2S,3S)-1-bromo-2,3-O-isopropylidene-cyclopent-4-ene building block starting from inexpensive methyl α-D-galactopyranoside. This synthesis is described in detail in the supplementary information.

### Q-base incorporation

For incorporation of Q-base at position 34, unlabeled or fluorescein labelled sptRNA^Asp^, was incubated with 100 fold molar excess of free Q base and under presence of 0.5 µM purified his-hTGT (100 mM HEPES pH 7.5, 20 mM MgCl_2_, 5 mM DTT). After four hours the reaction was precipitated with ethanol and pelleted RNA was resolubilized in H_2_O. Unreacted free Q-base was removed by desalting using Zeba spin desalting columns 7 K MWCO (ThermoFisher Scientific). Q-incorporation was confirmed by a Q-specific band-shift, a method previously described^27^, using denaturing 10% polyacrylamide gels containing urea and 0.5% (w/v) 3-aminophenylboronic acid (Sigma Aldrich).

### tRNA labelling and affinity measurements

5 nmol tRNA were incubated 50 min in 400 µL volume, containing 100 mM NaOAc pH5.5, 2.5 mM KIO_4_ at 4 °C. After ethanol precipitation the labelling reaction was carried out in 100 mM NaOAc pH 5.5 with 1 mM fluorescein-5-thiosemicarbazide (Sigma Aldrich). RNA was again ethanol precipitated and dissolved in H_2_O. Unreacted fluorophore was removed using Zeba spin desalting columns (ThermoFisher Scientific). spDnmt2 was desalted into assay buffer 20 mM Tris/HCl, 50 mM NaCl prior to the experiment. Anisotropy measurements were performed using a Fluoromax III (Horiba Jobin Yvon) fluorimeter and data was evaluated with FluorEssence software (Horiba Jobin Yvon).

### Methyl transferase assay

spDnmt2 kinetics were investigated using a methyltransferase assay kit with colorimetric detection (Cayman Chemicals). The assay is based on a previously reported detection of formation of the methyltransferase reaction co-product SAH by an enzymatic cascade Dorgan *et al*.^28^. In brief the SAH produced by de-methylation during the Dnmt2 methyl transfer reaction is cleaved by the SAH nucleosidase producing adenine which then undergoes desamination by the adenine desaminase yielding hypoxanthine. Oxidation of this product by the xanthine oxidase produces ureate and hydrogen peroxide latter of which oxidizes the colorimetric agent 3,5-dichloro-2-hydroxybenzenesulfonic acid. This oxidation is monitored at 515 nm. For performing this assay we prepared the reaction mastermix according to the manufacturers instructions. All measurements were carried out using the identical reaction buffer (50 mM Tris/HCl pH 7.5, 50 mM NaCl) to eliminate potential influence of ion strength and pH differences. spDnmt2 was transferred into assay buffer using Zeba spin desalting columns 7 K MWCO (ThermoFisher Scientific) and the concentration was calculated from the absorbtion at 280 nm using the absorption coefficient of the used spDnmt2 construct (50880 M^−1^ cm^−1^). To check that the assay is conducted under initial rate conditions we measured methyltransferase activity in triplicates with 10 µM G34tRNA^Asp^ for 0.5 µM and 0.125 µM enzyme yielding a ΔA_280_/min of 0.01070 ± 0.00014 and 0.00267 ± 0.00021 respectively after background subtraction. All measurements shown in Fig. 1 were carried out with enzyme derived from the identical purification. 6 µL of the desired tRNA concentration were added to 60 µL of the mastermix, mixed and preincubated at 20 °C shortly before measurement 3 µL of spDnmt2. 1 µM enzyme final were used for measurements with G34tRNA^Asp^ and 0.5 µM final spDnmt2 concentration for measuring enzyme activity on Q34tRNA^Asp^ to stay within the linear range of the assay. The reaction mixture was mixed and transferred immediately into a quartz cuvette with 10 mM path length (Hellma) for measurement. Change of absorbtion at 515 nm was monitored over time. Surface treated low binding tips (nerbe plus) were used in all pipetting steps. using an Ultrospec 2100 pro UV/Vis spectrometer (GE Healthcare) until slowing down of the reaction is observed. ΔA_515nm_/min for each measurement was obtained by fitting an asymptote to the steepest climb using swift II software (Fisher Scientific). Molar activity was calculated using the extinction coefficient of the colorimetric agent (E_515nm_ = 26000 M^−1^ cm^−1^). Data were plotted with Origin software (OriginLab) and fitted with the Hill equation v = V_max_ * x^n^/(k^n^ + x^n^) with free n value.

### Crystallization

spDnmt2 was crystallized using sitting-drop vapor diffusion technique. spDnmt2 was supplemented with five-fold molar excess of SAH (Sigma Aldrich) and adjusted to 2 mg/mL protein concentration as determined with Bradford reagent (Bio-Rad). Equal volumes of protein containing solution and crystallization buffer (50 mM MES pH 6.5, 1% w/v PEG4000, 4 mM MgCl_2_) were mixed. Crystals were obtained after five to seven days at 4 °C. Crystals were cryo-protected by a stepwise increase of glycerol and PEG400 concentration to 15% (v/v) each.

### Data collection and molecular replacement and refinement

X-ray data collection was performed at 100 K; diffraction images were collected at Petra III beamline P13 at DESY, Hamburg^29^. Diffraction images were indexed, integrated and scaled using the XDS-package^30^. The structure of spDnmt2 in complex with SAH was solved by molecular replacement by PHASER^31^, as implemented in the CCP4 suite^32^, using the structure of the *Entamoebae histolytica* Dnmt2 homolog EhMeth (PBD-ID: 3QV2)^17^.

Model building was done with subsequent iterative cycles of automated refinement with PHENIX^33^ and manual model adjustments in Coot^34^. Crystallographic values are listed in Supplementary Table S1.

### RNA-Protein cross-linking

To reconstitute the tRNA-protein complex, 3.75 nmol Dnmt2 and 3.75 nmol tRNA^Asp^ were incubated in 100 µl 20 mM HEPES, 50 mM NaCl 2 mM DTT, pH 7.5 for 30 min on ice. One sample containing tRNA^Asp^ was UV-irradiated at 254 nm for 10 min, while a second non-irradiated sample was kept as a non-cross-linked control. Further sample processing was performed as described previously^35^. To generate peptides from RNA-complexed proteins, both trypsin and chymotrypsin were used as described in generating peptides from RNA cross-linked proteins^35^.

### Mass spectrometry (MS/MS) and MS data analysis

Prepared samples were loaded onto a self-packed C18 column, mounted to a Dionex Ultimate 300 UHPLC^+^ focused (Thermo Scientific): 3 µm pore size, 75 µm in diameter, 30 cm in length (Reprosil-Pur® 120C18-AQ, Dr. Maisch GmbH). Peptides were separated by reverse-phase chromatography on a 58 min multi-step gradient with a flow rate of 0.3–0.4 µL/min before entering the mass spectrometer (OT Fusion Lumos, Thermo Scientific). MS1 spectra were recorded in profile mode with a resolution of 120 k, whereas MS2 spectra were recorded in centroid mode with a resolution of 30 k. The isolation window was set to 1.6 m/z and the dynamic exclusion was set to 9 s. Raw data of RNA-protein hetero-conjugates were analysed and manually validated using the OpenMS pipeline RNPxl and OpenMS TOPPASViewer^35^.

### Model preparation for use in Rosetta

The crystal structure of spDnmt2 monomer was subjected to an all-atom refinement with all-heavy-atom constraints in the Rosetta force field using the default Rosetta Relax protocol^36^. Running relax with side-chain and backbone coordinate constraints minimizes Rosetta energy by searching the local conformational space in order to remove clashes and rotamers with unfavorable Rosetta energy, while keeping all non-hydrogen atoms as close as possible to their starting positions in the crystal structure. The relaxation was carried out 20 times and the structure with the lowest energy was selected for *in silico* docking experiments. tRNA models were extracted from crystal structures (PDB ids: 1VTQ, 1ASZ, 1QF6, 4WJ3, 4WT8) of both unbound and protein-bound tRNA forms and thus representing different conformations of the molecule. These models were converted to a format suitable for Rosetta (all modified bases were replaced by unmodified ones).

### Protein-RNA docking experiments

Docking experiments were performed in Rosetta^37^ using the RosettaDock application optimized for predicting RNA-Protein complexes^38^. The used docking protocol was composed of two search stages: low resolution and high resolution. The low resolution stage uses a coarse-grained representation of the ligand and receptor (the side chains are replaced by a single unified pseudo-atom) making it possible to quickly sample the search space for the docking partners. The subsequent high resolution stage rebuilds the all-atom partners from the low resolution candidates to perform the full atom mode refinement including rotamer search and possibly loop optimization.

The low resolution stage was carried out for five tRNA models (PDB ids: 1VTQ, 1ASZ, 1QF6, 4WJ3, 4WT8). In order to fully sample the search space, 50.000 poses were generated for each tRNA model. Favorable initial docking orientations were identified based on the total Rosetta score as well as I_sc score (representing the energy of the interactions across the interface) and validated by the experimental crosslinks and distance constraints defined between the phosphate group of the modified nucleotide 38 and spDnmt2 active site residues. These active site residues have been identified based on superposition of the receptor (*sp*Dnmt2 crystal structure) with the crystal structure of Dnmt1 complexed with DNA^18^. The best scoring pose (low resolution model) was obtained for three-dimensional structure of yeast tRNA-ASP, PDB id: 1VTQ. This low resolution model has been subjected to the high resolution docking protocol (10.000 poses). Due to some short non-bonded interactions between the docked tRNA model and the receptor molecule, the high resolution docking step resulted in a positional shift of the tRNA molecule away from the active site (rmsd calculated for all phosphate atoms ~3 Å). In order to obtain a complete atomic model of the docked spDnmt2-tRNA complex satisfying both the experimental crosslinks and positional constraints (nucleotide 38 should be positioned close to the flipped nucleotide in the Dnmt1-DNA complex structure), we superimposed the 1VTQ structure onto the best low resolution model, thus skipping the high resolution docking step. Modeling of the flipped 38 nucleotide as well as building queuing modification at position 34 was performed in Coot.

### Data availability

Coordinates and structure factors have been deposited within the Protein Data Bank (PDB code 6FDF). All other datasets generated during the current study are available from the corresponding author on reasonable request.

## Electronic supplementary material

Supplementary Information
