# Supplementary material for: Structural insights into the stimulation of S. pombe Dnmt2 catalytic efficiency by the tRNA nucleoside queuosine
Source: Sci Rep. 2018 Jun 11;8:8880. doi: 10.1038/s41598-018-27118-5 (PMC5995894; doi:10.1038/s41598-018-27118-5)
Supplement: Supplementary file 1 — Supplementary Information [file 41598_2018_27118_MOESM1_ESM.pdf]

Supporting information

Title

Structural insights into the stimulation of *S.pombe* Dnmt2 catalytic efficiency by the tRNA nucleoside queuosine

Authors

Sven Johannsson[1], Piotr Neumann[1], Alexander Wulf[2], Luisa M. Welp[2], Hans-Dieter Gerber[3], Matthias Krull[4], Ulf Diederichsen[4], Henning Urlaub[2,5] and Ralf Ficner[1*]

Author affiliations

[1] Department of Molecular Structural Biology, Institute of Microbiology and Genetics, GZMB, Georg-August-University Göttingen, 37077 Göttingen, Germany

[2] Bioanalytical Mass Spectrometry Research Group, Max Planck Institute for Biophysical Chemistry, Am Faßberg 11, 37077 Göttingen, Germany;

[3] Institut für Pharmazeutische Chemie der Philipps-Universität Marburg, Marbacher Weg 6, 35032 Marburg, Germany.

[4] Institut für Organische und Biomolekulare Chemie, Georg-August-Universität Göttingen, Tammannstrasse 2, 37077, Göttingen, Germany.

[5] Bioanalytics, Department of Clinical Chemistry, University Medical Center Göttingen, Robert-Koch-Straße 40, 37075 Göttingen, Germany

* To whom correspondence should be addressed. Tel: +49 551 3914072; Email: rficner@uni-goettingen.de

Supplementary Table S1.

Crystallographic statistics of the spDnmt2 crystal structure.

**Data Collection**	
X-ray source	Synchrotron (PETRA III, P13)
Detector	Dectris Pilatus 6M-F
Wavelength (Å)	1.033
Space group	C2
Cell dimensions	
a, b, c (Å)	153.82, 114.78, 113.76
α, β, γ (°)	90.00, 131.05, 90.00
Resolution (Å)	40.80–1.70 (1.80-1.70)
R_{meas} (%)	3.5 (59.8)
I/σ(I)	20.63 (2.69)
$CC_{1/2}$	99.9 (81.3)
Completeness (%)	99.0 (90.3)
Redundancy	3.49 (3.57)
**Refinement**	
Resolution (Å)	30.0–1.7
No. reflections	162230
R_{work} (%)	17.39
R_{free} (%)	19.48
No. of atoms	
Protein	10490
Water	832
Ligand	170
Ions	0
B-factors (Å^2)	
Protein	44.3
Ligand	51.0
Water and other small molecules	49.6
R.m.s. deviations	
Bond lengths (Å)	0.005
Bond angles (°)	0.797
MolProbity analysis	
Ramachandran favoured (%)	96.94
Ramachandran outliers (%)	0.00
Rotamer outliers (%)	1.80
Clashscore	3.78

Supplementary Table S2.

Molar activity of spDnmt2 of the indicated final concentration of either G34tRNA^{Asp} (left) or Q34tRNA^{Asp}. Activity in U per enzyme concentration is calculated as mean values from three independent replicates. U is defined as substrate conversion in µmol/min. SD indicates standard deviation. Figure 1 represents a graphical representation of these values.

µM G34tRNA^{Asp}	U/µmol	SD
49.304	1.222	0.010
32.870	1.122	0.054
16.435	0.715	0.035
12.326	0.578	0.019
8.217	0.369	0.006
6.574	0.283	0.037
4.930	0.187	0.004
2.465	0.069	0.004

µM Q34tRNA^{Asp}	U/µmol	SD
23.212	2.078	0.012
15.475	1.958	0.023
7.737	1.532	0.044
5.803	1.163	0.004
4.836	0.999	0.032
3.869	0.878	0.036
2.321	0.491	0.018
1.161	0.296	0.008

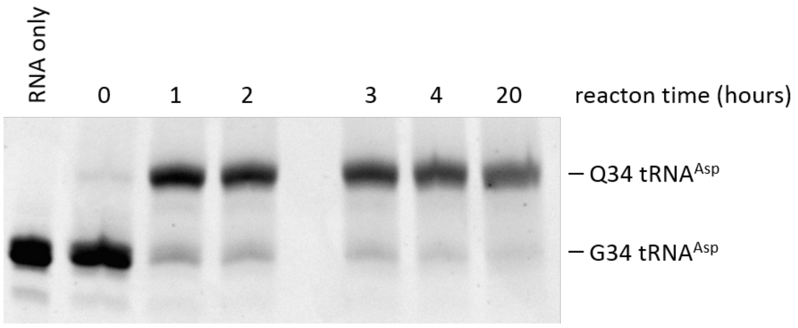

Supplementary Figure S1. Incorporation of queuine into tRNA^{Asp} by human TGT

tRNA^{Asp} was incubated with free queuine and TGT samples were taken from the reaction after addition of recombinant TGT at the indicated time and resolved on 3-phenylboronic acid containing denaturing 10% polyacrylamide gel.

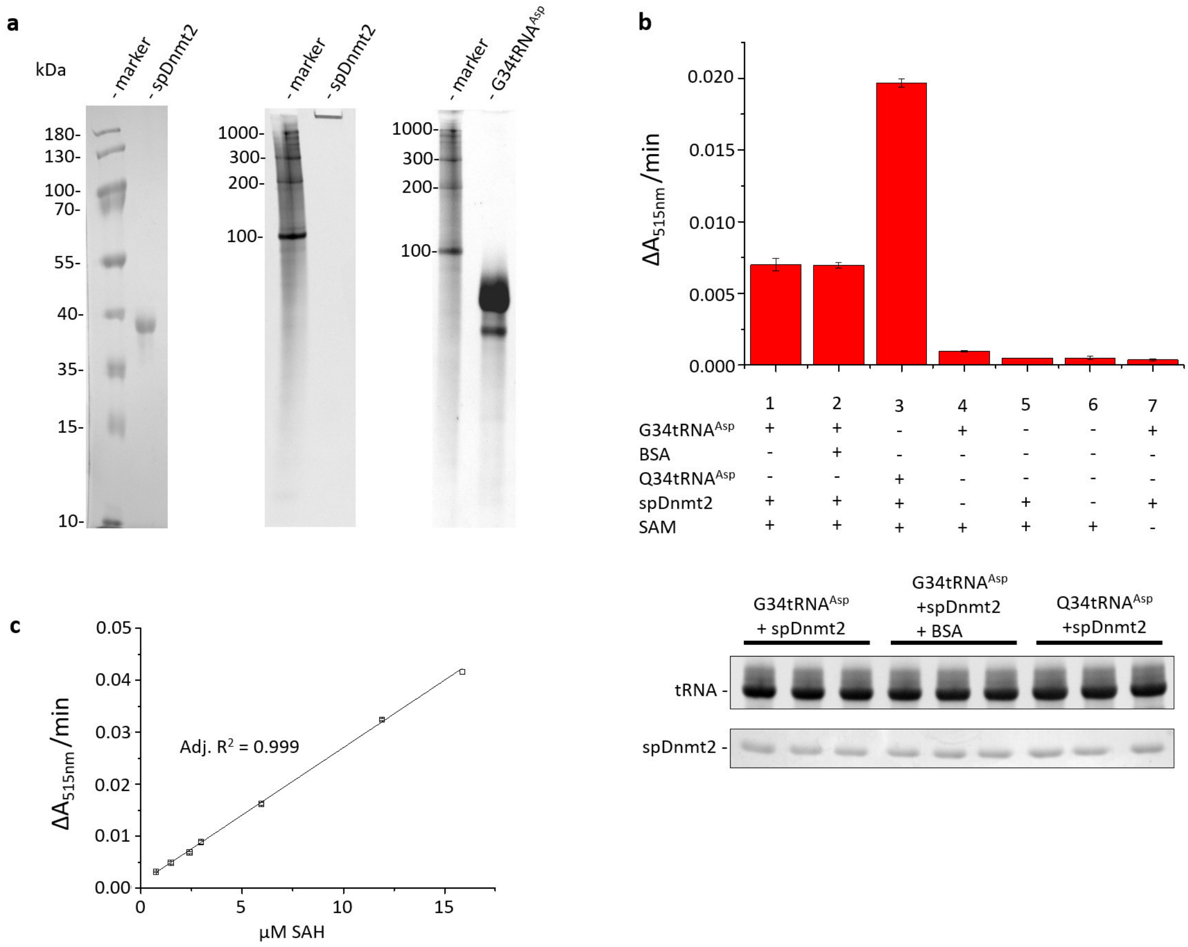

Supplementary Figure S2. Methyltransferase assay supporting data

(**a**) (right) Denaturing gel analysis of spDnmt2 as the enzyme is used in the assay. The left lane received 5 µL Prestained Protein Ladder (Fermentas). Sizes of bands in kilo Daltons are indicated on the left. 1 µg purified spDnmt2 protein was loaded in the right lane. (middle) Denaturing 8 M Urea containing gel with 10 % Polyacrylamide. Left lane received 5µL of Ribo Ruler Low Range RNA ladder (Fermentas), which equals to 175µg per band. Band sizes in bases are indicated on the left. 5µg purified spDnmt2 was loaded in the right lane. (right) Denaturing 8 M Urea containing gel with 10 % Polyacrylamide. Left lane received 5µL of Ribo Ruler Low Range RNA ladder (Fermentas). Purified G34tRNAAsp was loaded in the right lane. (**b**) The methyltransferase assay was performed as described in the materials but with different combinations of components. Indicated measurements received 5.5 µM G34tRNA^{Asp} Q34tRNA^{Asp} and/or 1 µM spDnmt2 final concentrations. Change in absorbance was measured at 515 nm over time. Every measurement was carried out as three

independent triplicates. Error bars indicate standard deviation. No change in reaction velocity was observed for spDnmt2 activity on G34tRNA^{Asp} when reaction tubes were coated for several minutes with 0.1 M BSA prior to mixing of the components. spDnmt2 exhibits almost three-fold higher activity on the queuine modified compared to the unmodified tRNA at this substrate concentration. Below, equal use of tRNA and Dnmt2 in reaction triplicates is shown. Samples were taken after monitoring the reaction and mixed in equal amounts with 2x RNA loading dye or 2x SDS loading dye. equal volumes of 8 µL per sample or 10 µL per lane were loaded on a denaturing 10 % polyacrylamide Urea gel or 12.5 % polyacrylamide SDS-PAGE gel respectively. The Urea and SDS containing gels were stained with GelRed (Biotinum) or comassie respectively for visualization. (**c**) Overview of increasing ΔA_{515nm} /min with increasing concentrations of SAH. Reaction mixture did not receive RNA or protein but the indicated final concentration of SAH. Linear regression shows the linear dependency of change in absorbtion at 515 nm with increasing concentration of SAH within the measured assay range.

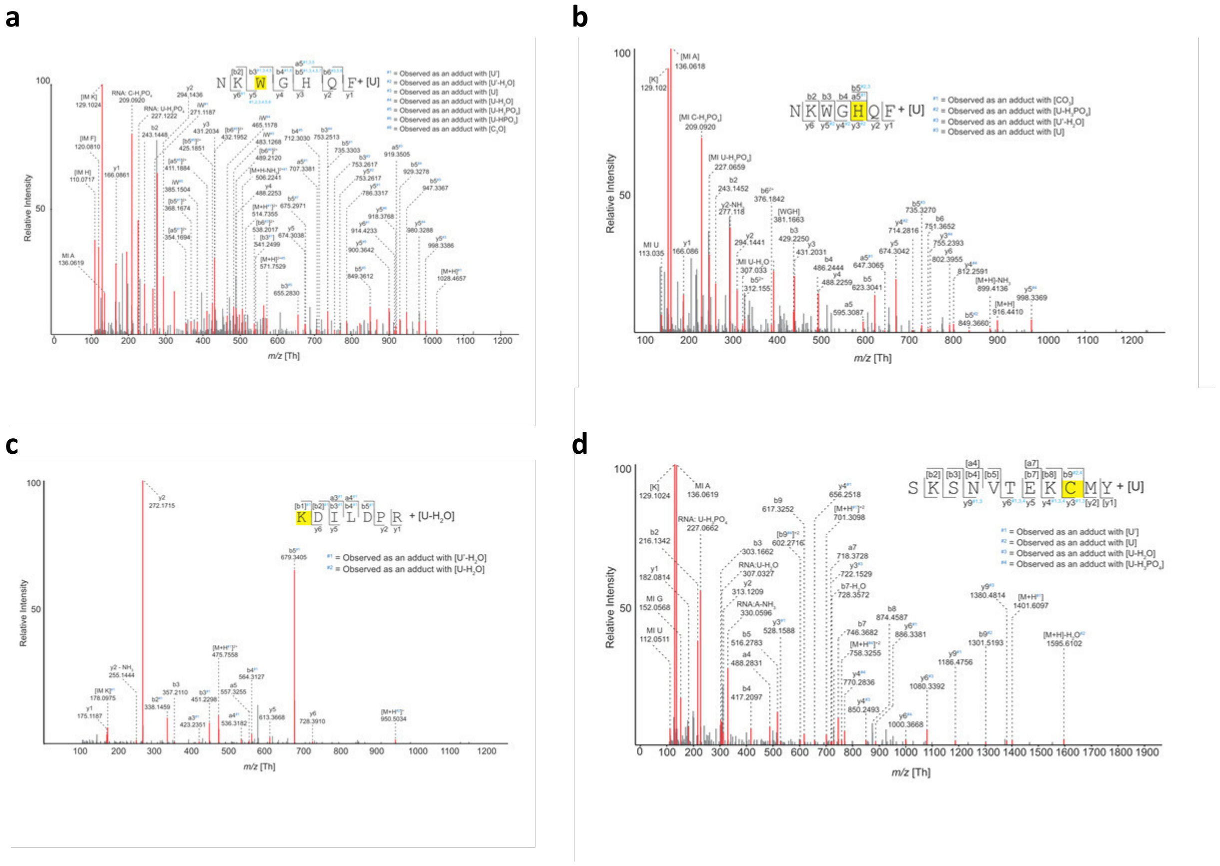

Supplementary Figure S3. MS/MS spectra of RNA (oligo) nucleotides cross-linked to spDnmt2 peptides identified for the tRNA^{Asp}-Dnmt2 complex

(**a**) The chymotryptic heptapeptide N_{219}-F_{225} cross-linked to a uracil nucleotide. The identification of both shifted y-ions and multiple b-ions and in addition to a critical y5/ b3 shifted ion pair, unambiguously assigns the location of the cross-link to W_{221} (highlighted in yellow). **(b)** The same chymotryptic heptapeptide as in (a) was found to be cross-linked to a uracil nucleotide. In this case, H_{223} is the cross-linked amino acid as revealed by the shifted y3, y4, y5 and b5 fragment ions. (**c**) The tryptic heptapeptide K_{91}-R_{97} was also found to be cross-linked to a uracil nucleotide, as identified by an almost complete shifted b-ion series starting at b1 in addition to shifted a3 and a4 ions ions. K_{91} was identified to be the cross-linking amino acid. (**d**) For the chemotryptic peptide S_{295}-$Y_{305,}$ a cysteine residue was identified to be cross-linked to uracil. Shifted y-ions originating at the y3 ion, and the shifted b9-ion identify C_{303} as the cross-linked amino acid. Ions with a mass shift of $\#^1$, $\#^2$, $\#^3$, $\#^4$, $\#^5$, $\#^6$ and $\#^7$ correspond to the cross-linked nucleotides U', U'-H_2O, U, U-H_2O, U-H_3PO_4, U-HPO_3 and CO_3, respectively. U: Uracil (324.04 Da); U': nucleobase of U (112.02 Da).

Synthesis of queuine

The reported synthetic routs for the preparation of queuine[1-5] require a huge synthetic effort and are based on expensive or toxic reagents. Furthermore, current syntheses afford stereoselective conversions. Gerber and Klebe reported a queuine synthesis based on fragment coupling of 7-aminoethyl-7-deazaguanine (preQ) and (1R,2S,3S)-1-bromo-2,3-O-isopropylidene-cyclopent-4-ene building blocks[6]. Based on this strategy, herein a new route to the (1R,2S,3S)-1-bromo-2,3-O-isopropylidene-cyclopent-4-ene building block is described starting from inexpensive methyl α-D-galactopyranoside. Thereby, formation of the chiral centers by stereoselective reactions and usage of toxic pyridinium chlorochromate was avoided. α-D-galactopyranoside already contains the configurational pattern for the hydroxyl groups at positions 2, 3 and 4 of the cyclopentene building block used in the Q-base synthesis. The α-D-galactopyranoside configuration was conserved, suitable protecting groups were applied and enantioselective reactions made expendable.

As outlined in supplementary figure S3 the hydroxyl groups at C2, C3 and C4 of α-D-galactopyranoside (**1**) were protected as acetal and silyl ether, respectively, and the primary alcohol was converted into the iodide. Reduction of sugar **3** at C5 and C6 with zinc provided the terminal olefin and the aldehyde in intermediate **4**. After conversion of the aldehyde to a second terminal olefin, ring closing metatheses yielded cyclopenteneol **7**, which was then brominated to give (1R,2S,3S)-1-bromo-2,3-O-isopropylidene-cyclopent-4-ene (**8**) as the desired building block.

Compounds **2**, **3**, and **4** were synthesized following slightly modified protocols from Moynihan *et al.*[7] Compound **7** was synthesized following a modified protocol from Smith *et al.*[8] Compounds **8**, **10**, and **12** were synthesized following a protocol developed by Gerber *et al.*[6].

General Reagent Information

All reagents, including anhydrous solvents, were purchased from commercial suppliers (*ABCR, Acros-Organics, Alfa Aesar, Fisher Scientific, Merck, Roth, Sigma-Aldrich, TCI* and *VWR*) were used without further purification. Technical solvents were distilled prior to use. Water was purified using a Simplicity water purification system from Millipore.

General Experimental Methods

NMR spectra were recorded at *Varian* instruments *(Mercury 300, Mercury VX 300, Unity 300, VNMRS-300*) and *Bruker instruments* (*Avance III 400, Avance III HD 400*) using chloroform-*d* or methanol-d_4 as the internal standard. Chemical shifts are quoted in ppm. Multiplicities are abbreviated as follows: s = singlet, d = doublet, dd = doublet of doublets, t = triplet, dt = doublet of triplets, m = multiplet, dm = doublet of multiplets.

High resolution ESI spectra were obtained with a *Bruker APEX-Q IV 7T* spectrometer. The values are given as m/z relation.

Reactions were monitored by thin-layer chromatography (**TLC**) carried out on aluminium backed plates of silica gel 60 F_{254} (layer thickness: 0.20 mm) from *Merck*. Spots were detected by fluorescence quenching at 254 nm or dying with $KMnO_4$-solution. Purification by **flash chromatography** was conducted using *Merck* Silica Gel 60 (particle size: 40-62 μm).

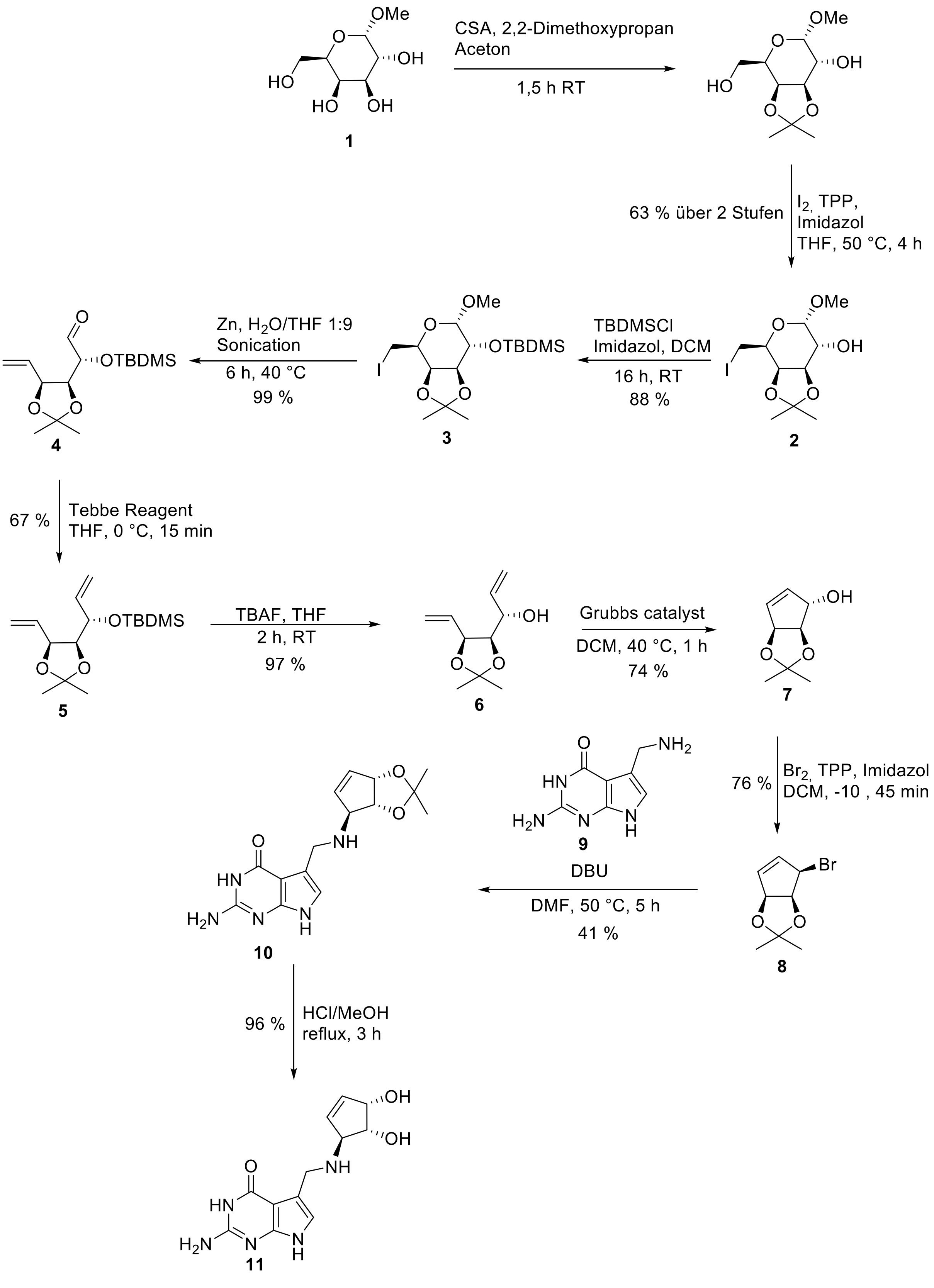

Supplementary Figure S4. Synthetic route to queuine (11)

Methyl 6-deoxy-6-iodo-3,4-O-isopropylidene-α-ᴅ-galactopyranoside (2)

To a stirred suspension of methyl α-D-galactopyranoside (**1**) (25 g, 129 mmol, 1.0 eq.) in acetone (450 mL), 2,2-dimethoxypropane (39,5 mL, 233 mmol, 2.5 eq.) and camphor sulfonic acid (5 mol%, 1.5 g, 6.45 mmol) were added and the resulting solution was stirred for 2 h at ambient temperature. After addition of NEt_3 (3 mL) the mixture was evaporated *in vacuo* and the resulting residue was resolved in THF (900 mL). Triphenylphosphine (50.75 g, 194 mmol, 1.5 eq.) and imidazole (17.6 g, 258 mmol, 2.0 eq.) were added and the solution was heated to 50 °C. Subsequently a solution of iodine (49.2 g, 194 mmol, 1.5 eq.) in THF (180 mL) was added dropwise and the solution was stirred for 2.5 h at 50 °C. The solution was cooled to ambient temperature and quenched with aqueous $Na_2S_2O_3$ solution (10%, 600 mL). The layers were separated, and the aqueous layer was extracted with EtOAc (3 x 200 mL). The combined organic layers were washed with sat. aqueous NaCl solution (2 x 200 mL), dried over $MgSO_4$ and the solvents were evaporated *in vacuo*. The crude product was purified by column chromatography on silica gel using pentane/EtOAc 1:1. The desired product **2** (27.9 g, 81.1 mmol, 63% over two steps) was obtained as a white solid.

^{1}H-NMR ($CDCl_3$, 300 MHz): δ = 4.68 (d, *J* = 3.9 Hz, 1 H, *H*-1), 4.25-4.17 (m, 2 H, *H*-3, *H*-4), 4.08-4.00 (m, 1 H, *H*-5), 3.81-3.75 (m, 1 H, *H*-6), 3.43 (s, 3 H, OCH_3), 3.30-3.17 (m, 2 H, *H*-6, *H*-6'), 1.40 (s, 3 H, $C(CH_3)_2$), 1.26 (s, 3 H, $C(CH_3)_2$) ppm.

^{13}C-NMR ($CDCl_3$, 126 MHz): δ = 109.7 ($C(CH_3)_2$), 98.2 (*C*-1), 75.8 (*C*-3), 73.5 (*C*-4), 69.4 (*C*-5), 68.6 (*C*-2), 55.6 (OCH_3), 27.4 ($C(CH_3)_2$), 25.7 ($C(CH_3)_2$), 2.8 (*C*-6) ppm.

MS (ESI): **m/z** (%) = 367.0 (100) $[M+Na]^+$.

HRMS (ESI): calc. for $C_{10}H_{17}IO_5Na$ $[M+Na]^+$: 367.0013, found 367.0017.

Methyl 6-deoxy-6-iodo-3,4-O-isopropylidene-2-O-tertbutyldimethylsilyl-α-ᴅ-galactopyrano-side (3)

Under argon atmosphere iodide **2** (10.0 g, 29.1 mmol, 1.0 eq.), imidazole (2.38 g, 34.9 mmol, 1.2 eq.) and TBDMSCl (5.26 g, 34.9 mmol, 1.2 eq.) were dissolved in dry DCM (120 mL) and the solution was stirred for 16 h at ambient temperature. The reaction was quenched with water (100 mL). The layers were separated, and the

organic layer was washed with water (2 x 60 mL) and sat. aqueous NaCl solution (60 mL), dried over $MgSO_4$ and the solvents were evaporated *in vacuo*. The crude product was purified by column chromatography on silica gel using pentane/EtOAc 95:5. The desired product **3** (11.35 g, 24.8 mmol, 85%) was obtained as clear oil.

^{1}H-NMR ($CDCl_3$, 300 MHz): δ = 4.59 (d, *J* = 3.6 Hz, 1 H, *H*-1), 4.29 (dd, *J* = 5.7, 2.4 Hz, 1 H, *H*-4), 4.19-4.08 (m, 2 H, 3-H, *H*-3), 3.74 (dd, *J* = 7.2, 3.6 Hz, 1 H, *H*-2), 3.44 (s, 3 H, OCH_3), 3.41-3.29 (m, 2 H, *H*-6, *H*-6'), 1.48 (s, 3 H, $C(CH_3)_2$), 1.34 (s, 3 H, $C(CH_3)_2$), 0.98 (s, 9 H, $SiC(CH_3)_3$), 0.10 (s, 3 H, $Si(CH_3)_2$), 0.08 (s, 3 H, $Si(CH_3)_2$) ppm.

^{13}C-NMR ($CDCl_3$, 126 MHz): δ = 109.2 ($C(CH_3)_2$), 100.2 (*C*-1), 76.9 (*C*-3), 74.0 (*C*-4), 71.2 (*C*-2), 68.6 (*C*-5), 55.8 (OCH_3), 28.1 ($C(CH_3)_2$), 26.2 ($C(CH_3)_2$), 25.8 ($SiC(CH_3)_3$), 18.1 ($SiC(CH_3)_3$), 2.85 (*C*-6), 4.58 ($Si(CH_3)_2$), 4.75 ($Si(CH_3)_2$) ppm.

MS (ESI): **m/z** (%) = 481.1 (100) $[M+Na]^+$.

HRMS (ESI): calc. for $C_{16}H_{31}IO_5SiNa$ $[M+Na]^+$: 481.0878, found 481.0878.

(2R,3S,4S)-3,4-O-Isopropylidene-2-O-tertbutyldimethylsilyl-hex-5-en-1-al (4)

Galactopyranoside **3** (32.6 g, 71.2 mmol, 1.0 eq.) and was dissolved in THF/H_2O (9:1, v,v, 600 mL) and zinc powder (46.5 g, 710 mmol, 10.0 eq.) was added. The suspension was stirred at 50 °C for 6 h. After cooling to ambient temperature, the suspension was filtered through a silica plug and washed with Et_2O (300 mL). The organic layer was washed with water (200 mL), sat. aqueous $NaHCO_3$ solution (200 mL) and sat. aqueous NaCl solution (200 mL), dried over $MgSO_4$ and the solvents were evaporated *in vacuo* to give the desired product **4** (21.2 g, 70.6 mmol, 99%) as yellowish oil.

^{1}H-NMR ($CDCl_3$, 300 MHz): δ = 9.67 (d, *J* = 0.9 Hz, 1 H, C*H*O), 5.92 (ddd, *J* = 17.3, 10.4, 6.8 Hz, 1 H, *H*-5), 5.35, (dt, *J* = 17.3, 1.5 Hz, 1 H, *H*-6), 5.21 (dt, *J* = 10.4, 1.5 Hz, 1 H, *H*-6'), 4.72 (t, *J* = 6.8 Hz, 1 H, *H*-4), 4.32 (dd, *J* = 6.8, 5.3 Hz, 1 H, *H*-3), 4.07 (dd, *J* = 5.3, 0.9 Hz, 1 H, *H*-2), 1.51 (s, 3 H, $C(CH_3)_2$), 1.35 (s, 1 H, $C(CH_3)_2$), 0.92 (s, 9 H, $SiC(CH_3)_3$), 0.09 (d, *J* = 1.8 Hz, 6 H, $Si(CH_3)_2$) ppm.

^{13}C-NMR ($CDCl_3$, 126 MHz): δ = 201.8 (*C*HO), 133.8 (*C*-5), 118.9 (*C*-6), 109.2 ($C(CH_3)_2$), 79.1 (*C*-3), 78.5 (*C*-4), 77.4 (*C*-2), 27.1 ($C(CH_3)_2$), 25.9 ($SiC(CH_3)_3$), 25.2 ($C(CH_3)_2$), 18.4 ($SiC(CH_3)_3$), -4.4 ($Si(CH_3)_2$), -4.7 ($Si(CH_3)_2$) ppm.

MS (ESI): **m/z** (%) = 323.2 (0.5) $[M+Na]^+$.

HRMS (ESI): calc. for $C_{15}H_{28}O_4SiNa$ $[M+Na]^+$: 323.1649, found 323.1648.

(2S,3S,4S)-3,4-O-Isopropylidene-2-O-tertbutyldimethylsilyl-hepta-1,5-diene (5)

Under an argon atmosphere aldehyde **4** (21.2 g, 70.6 mmol, 1.0 eq.) was dissolved in dry THF (200 mL) and the solution was cooled to 0 °C. TEBBE REAGENT (0.5 M in toluene, 200 mL, 100 mmol, 1.4 eq.) was added dropwise and the resulting solution was stirred at 0 °C for 30 min. Subsequently Et_2O (200 mL) was added and water (12 mL) was added dropwise. The resulting suspension was dried over $MgSO_4$, filtered and the residue was washed with Et_2O (100 mL). The solvents of the combined organic layers were evaporated *in vacuo* and the resulting crude product was purified by column chromatography on silica gel using pentane/EtOAc 95:5. The desired product **5** (14.1 g, 47.1 mmol, 67%) was obtained as an orange oil.

^{1}H-NMR ($CDCl_3$, 300 MHz): δ = 6.04-5.80 (m, 2 H, *H*-2, *H*-6), 5.41-5.12 (m, 4 H, *H*-1, *H*-7), 4.48-4.41 (m. 1 H, *H*-5), 4.22-4.14 (m, 1 H, *H*-4), 4.02-3.95 (m, 1 H, *H*-3), 1.50 (s, 3 H, $C(CH_3)_2$), 1.35 ($C(CH_3)_2$), 0.91 (s, 9 H, $SiC(CH_3)_3$), 0.09 (s, 3 H, $Si(CH_3)_2$), 0.07 (s, 3 H, $Si(CH_3)_2$) ppm.

^{13}C-NMR ($CDCl_3$, 126 MHz): δ = 136.9 (*C*-2), 134.8 (*C*-6), 118.7 (*C*-7), 116.6 (*C*-1), 108.7 ($C(CH_3)_2$), 82.0 (*C*-4), 79.2 (*C*-5), 72.7 (*C*-3), 28.0 ($C(CH_3)_2$), 26.0 ($SiC(CH_3)_3$), 25.7 ($C(CH_3)_2$), 18.6 ($SiC(CH_3)_3$), -4.4 ($Si(CH_3)_2$), -4.5 ($Si(CH_3)_2$) ppm.

MS (ESI): **m/z** (%) = 321.2 (14) $[M+Na]^+$.

HRMS (ESI): calc. for $C_{16}H_{30}O_3SiNa$ $[M+Na]^+$: 321.1856, found 321.1854.

(2S,3S,4S)-2-Hydroxy-3,4-O-isopropylidene-hepta-1,5-diene (6)

Diene **5** (13.6 g, 45.6 mmol, 1.0 eq.) was dissolved in THF (500 mL) and a solution of $TBAF\cdot 3H_2O$ (28.8 g, 91.2 mmol, 2.0 eq.) in THF (100 mL) was added dropwise. The solution was stirred for 2.5 h at ambient temperature. The solvent was evaporated *in vacuo* and the resulting crude product was purified by column chromatography on silica gel using pentane/EtOAc 5:1. The desired product **6** (8.12 g, 44.1 mmol, 97%) was obtained as yellow oil.

^{1}**H-NMR** ($CDCl_3$, 300 MHz): δ = 6.07-5.93 (m, 1 H, *H*-2), 5.90-5.77 (m, 1 H, *H*-6), 5.44-5.17 (m, 4 H, *H*-1, *H*-7), 4.63-4.55 (m, 1 H, *H*5), 4.16-4.04 (m, 2 H, *H*-4, *H*-3), 1.52 (s, 3 H, $C(CH_3)_2$), 1.38 (s, 3 H, $C(CH_3)_2$) ppm.

^{13}**C-NMR** ($CDCl_3$, 126 MHz): δ = 136.8 (*C*-2), 134.1 (*C*-6), 119.5 (*C*-7), 117.2 (*C*-1), 108.1 ($C(CH_3)_2$), 80.8 (*C*-4), 79.7 (*C*-5), 70.7 (*C*-3), 27.5 ($C(CH_3)_2$), 25.1 ($C(CH_3)_2$) ppm.

MS (ESI): **m/z** (%) = 207.1 (7) $[M+Na]^+$.

HRMS (ESI): calc. for $C_{10}H_{16}O_3Na$ $[M+Na]^+$: 207.0992, found 207.0992.

(1S,2S,3S)-1-Hydroxy-2,3-O-isopropylidene-cyclopent-4-ene (7)

Under an argon atmosphere diene **6** (8.12 g, 44.1 mmol, 1.0 eq.) and GRUBB'S CATALYST, 2ND GEN. (2.3 mol%, 848 mg, 1.0 eq.) were dissolved in dry DCM (200 mL) and the solution was stirred under reflux for 1 h. After cooling to ambient temperature, the solvent was evaporated *in vacuo* and the resulting crude product was purified by column chromatography on silica gel using pentane/EtOAc 2:1. The desired product **7** (5.2 g, 32.7 mmol, 74%) was obtained as brown oil.

^{1}**H-NMR** ($CDCl_3$, 300 MHz): δ = 6.00 (d, *J* = 5.7 Hz, 1 H, *H*-5), 5.88 (dd, *J* = 5.7, 2.0 Hz, 1 H, *H*-4), 5.27 (dd, *J* = 5.6, 1.0 Hz, 1 H, *H*-3), 4.76 (s, 1 H, *H*-2), 4.49 (d, *J* = 5.6 Hz, 1 H, *H*-1), 1.38 (s, 3 H, $C(CH_3)_2$), 1.33 (s, 3 H, $C(CH_3)_2$) ppm.

^{13}**C-NMR** ($CDCl_3$, 126 MHz): δ = 135.5 (*C*-5), 134.8 (*C*-4), 111.8 ($C(CH_3)_2$), 86.0 (*C*-3), 84.4 (*C*-2), 81.1 (*C*-1), 27.4 ($C(CH_3)_2$), 25.8 ($C(CH_3)_2$) ppm.

MS (ESI): **m/z** (%) = 179.1 (4) $[M+Na]^+$.

HRMS (ESI): calc. for $C_8H_{11}O_2BrNa$ $[M+Na]^+$: 179.0679, found 179.0678.

(1R,2S,3S)-1-Bromo-2,3-O-isopropylidene-cyclopent-4-ene (8)

Triphenylphosphine (16.1 g, 61.4 mmol, 2.0 eq.) was dissolved in DCM (300 mL) and cooled to -10 °C and a solution of bromine (3.1 mL, 61.4 mmol, 2.0 eq.) in DCM (20 mL) was added dropwise. The resulting solution was added slowly to a solution of alcohol **7** (4.8 g, 30.7 mmol, 1.0 eq.) and imidazole (2.3 g, 33.8 mmol, 1.1 eq.) in DCM

(150 mL) at -10 °C. The reaction mixture was stirred at -10 °C for 45 min, then allowed to reach ambient temperature and Et_2O (300 mL) was added. The resulting organic solution was washed with water (2 x 300 mL) and sat. aqueous NaCl solution (300 mL), dried over $MgSO_4$ and the solvents were evaporated *in vacuo*. The residue was dissolved in DCM (10 mL) and hexane (500 mL) was added. The resulting suspension was filtrated, and the filtrate was concentrated *in vacuo*. The residue was suspended in hexane (150 mL) and filtrated. The filtrate was concentrated *in vacuo* and the resulting crude product purified by column chromatography on silica gel using pentane/EtOAc 95:5. The desired product **8** (5.1 g, 23.3 mmol, 76%) was obtained as colorless oil.

^{1}H-NMR ($CDCl_3$, 300 MHz): δ = 6.01-5.97 (m, 1 H, *H*-5), 5.97-5.93 (m, 1 H, *H*-4), 5.32 (d, *J* = 5.4 Hz, 1 H, *H*-3), 4.96 (d, *J* = 5.4 Hz, 1 H, *H*-2), 4.86-4.82 (m, 1 H, *H*-1), 1.38 (s, 3 H, $C(CH_3)_2$), 1.34 (s, 3 H, $C(CH_3)_2$) ppm.

^{13}C-NMR ($CDCl_3$, 126 MHz): δ = 135.1 (*C*-5), 134.2 (*C*-4), 112.2 ($C(CH_3)_2$), 86.0 (*C*-3), 84.1 (*C*-2), 54.5 (*C*-1), 27.5 ($C(CH_3)_2$), 26.3 ($C(CH_3)_2$) ppm.

MS (ESI): **m/z** (%) = 241.0 (7) $[M+Na]^+$.

HRMS (ESI): calc. for $C_8H_{11}O_2BrNa$ $[M+Na]^+$: 240.9835, found 240.9833.

2',3'-O-Isopropylidene-queuine (10)

Under an argon atmosphere pre-Q (**9**) (1.0 g, 4.0 mmol, 1.0 eq.) was dissolved in DMF (15 mL), DBU (6.0 mL, 40.0 mmol, 10.0 eq.) was added and the solution was stirred at 50 °C for 15 min. A solution of bromine **8** (1.1 g, 4.8 mmol, 1.2 eq.) in DMF (3 mL) was added dropwise and the solution was stirred at 50 °C for 5 h. After cooling to ambient temperature, the solvent was evaporated *in vacuo* and the residue was purified by column chromatography on silica gel using EtOAc/MeOH/NEt_3 3:1:0.1. The resulting crude product was suspended in $CHCl_3$ (100 mL) and stirred for 10 min. The solid components were separated by filtration and suspended in methanol (30 mL). The suspension was filtrated, and the filtrate was concentrated *in vacuo* to yield the desired product **10** (581 mg, 1.64 mmol, 41%) as a slightly yellow solid.

^{1}H-NMR (CD_3OD, 300 MHz): δ = 6.90 (s, 1 H, *H*-8), 6.30 (dt, *J* = 5.9 Hz, 1.7 Hz, 1 H, *H*-5'), 6.01 (dm, *J* = 5.9 Hz, 1 H, *H*-4'), 5.36 (dm, *J* = 5.8 Hz, 1 H, *H*-3'), 4.94 (d,

J = 5.7 Hz, 1 H, *H*-2), 4.49-4.29 (m, 3 H, *H*-1', H_2-10), 1.37 (s, 3 H, C(CH_3)$_2$), 1.36 (s, 3 H, C(CH_3)$_2$) ppm.

MS (ESI): **m/z** (%) = 318.2 (100) $[M+H]^+$, 340.2 (4) $[M+Na]^+$.

HRMS (ESI): calc. for $C_{15}H_{20}N_5O_3$ $[M+H]^+$: 318.1561, found 318.1562.

Queuine (11)

2',3'-O-Isopropylidene-queuine (**10**) (581 mg, 1.64 mmol, 1.0 eq.) was dissolved in methanol (60 mL) and conc. HCl solution (5 mL) was added. The solution was stirred under reflux for 2 h. After cooling to ambient temperature, the resulting suspension was concentrated *in vacuo* to yield queuine (**11**) (544 mg, 1.57 mmol, 96%) as a slightly yellow solid.

^{1}H-NMR (CD_3OD, 300 MHz): δ = 7.04 (s, 1H, *H*-8), 6.28 (dt, *J* = 6.3, 2.2 Hz, 1 H, *H*-5'), 6.10 (dd, *J* = 6.3, 1.7 Hz, 1 H, *H*-4'), 4.71-4.65 (m, 1 H, *H*-3'), 4.55-4.25 (m, 4 H, *H*-2', *H*-1', H_2-10) ppm.

^{13}C-NMR ($CDCl_3$, 126 MHz): δ = 161.8 (*C*-6), 152.6 (*C*-2), 146.6 (*C*-4), 138.9 (*C*-4'), 129.8 (*C*-5'), 121.0 (*C*-7), 109.8 (*C*-8), 99.9 (*C*-5), 74.7 (*C*-3'), 74.0 (*C*-2'), 87.8 (*C*-1'), 42.6 (*C*-10) ppm.

MS (ESI): **m/z** (%) = 278.1 (95) $[M+H]^+$, 276.1 (100) $[M-H]^-$, 312.1 (75) $[M+Cl]^-$.

HRMS (ESI): calc. for $C_{12}H_{15}N_5O_3Na$ $[M+Na]^+$: 300.1067, found 300.1063.

References

1 Akimoto, H. *et al.* Queuine analogues. Their synthesis and inhibition of growth of mouse L5178Y cells in vitro. *J Med Chem* **29**, 1749-1753 (1986).

2 Akimoto, H., Imamiya, E., Hitaka, T., Nomura, H. & Nishimura, S. Synthesis of Queuine, the Base of Naturally-Occurring Hypermodified Nucleoside (Queuosine), and Its Analogs. *Journal of the Chemical Society-Perkin Transactions 1*, 1637-1644, doi:DOI 10.1039/p19880001637 (1988).

3 Klepper, F., Polborn, K. & Carell, T. Robust synthesis and crystal-structure analysis of 7-cyano-7-deazaguanine (PreQ(0) base) and 7-(aminomethyl)-7-deazaguanine (PreQ(1) base). *Helvetica Chimica Acta* **88**, 2610-2616, doi:DOI 10.1002/hlca.200590201 (2005).

4 Barnett, C. J. & Grubb, L. M. Total synthesis of Q Base (Queuine). *Tetrahedron* **56**, 9221-9225, doi:Doi 10.1016/S0040-4020(00)00895-4 (2000).

5 Brooks, A. F., Garcia, G. A. & Showalter, H. D. H. A short, concise synthesis of queuine. *Tetrahedron Letters* **51**, 4163-4165, doi:10.1016/j.tetlet.2010.06.008 (2010).

6 Gerber, H. D. & Klebe, G. Concise and efficient syntheses of preQ1 base, Q base, and (ent)-Q base. *Org Biomol Chem* **10**, 8660-8668, doi:10.1039/c2ob26387d (2012).

7 Moynihan, L., Chadda, R., McArdle, P. & Murphy, P. V. Allylic Azide Rearrangement in Tandem with Huisgen Cycloaddition for Stereoselective Annulation: Synthesis of C-Glycosyl Iminosugars. *Organic Letters* **17**, 6226-6229, doi:10.1021/acs.orglett.5b03209 (2015).

8 Smith, A. B., Sperry, J. B. & Han, Q. Syntheses of (-)-oleocanthal, a natural NSAID found in extra virgin olive oil, the (-)-deacetoxy-oleuropein aglycone, and related analogues. *Journal of Organic Chemistry* **72**, 6891-6900, doi:10.1021/jo071146k (2007).

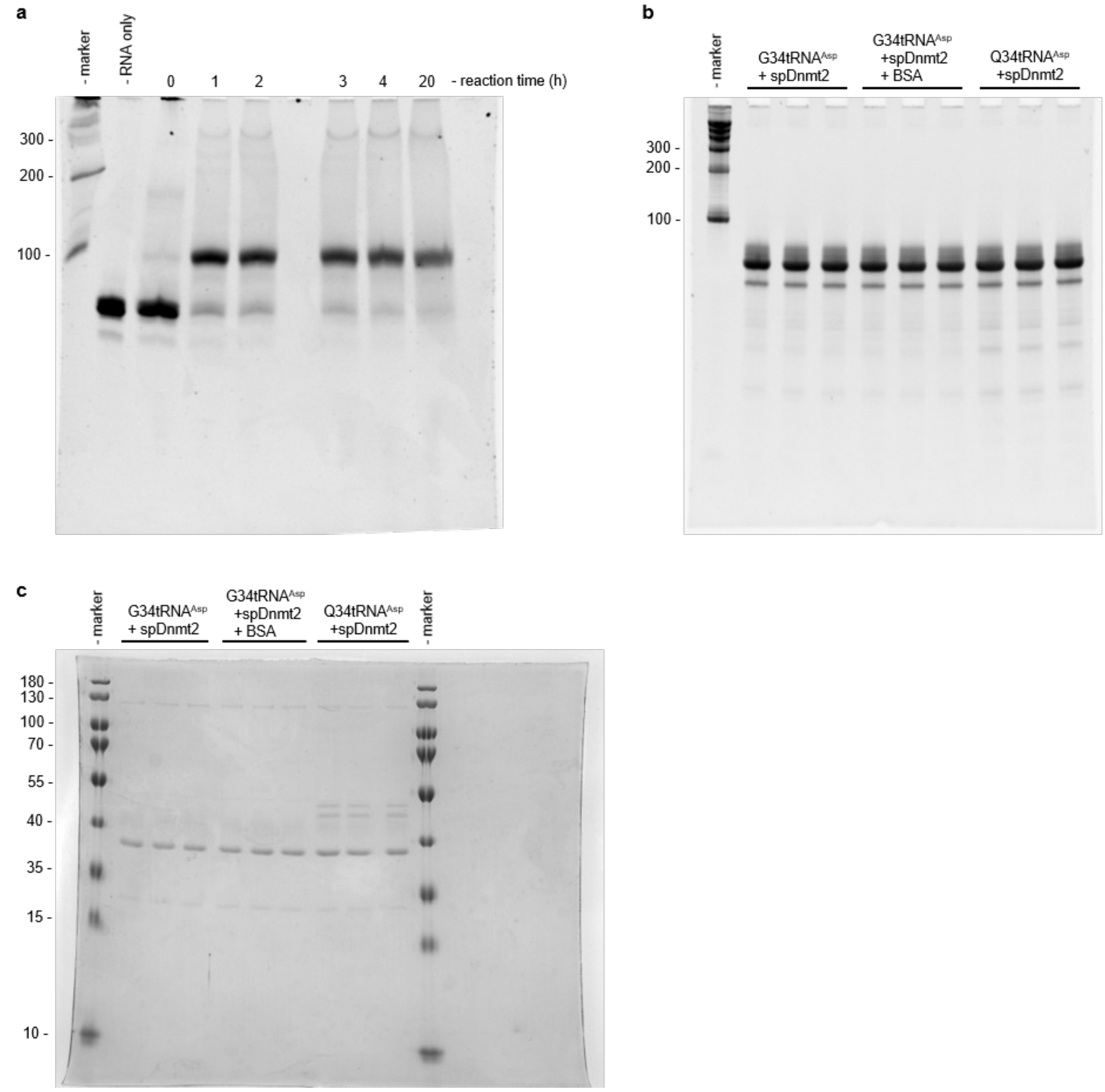

Supplementary Figure S5. Uncropped gel images

a Original image of the 3-phenylboronic acid containing denaturing 10% polyacrylamide gel shown in supplementary Figure S1. Ribo Ruler low range ladder (Fermantas) was used as a marker. Marker band-sizes are indicated as bases. **b** Original, image of the denaturing 10% polyacrylamide gel stained with GelRed (Biotinum) shown in supplementary Figure S2 part b. Ribo Ruler low range ladder (Fermantas) was used as a marker. Marker band-sizes are indicated as bases. **c** Original, image of the 12,5% SDS-polyacrylamide gel stained with Coomassie shown in supplementary Figure S2 part b. Prestained Protein Ladder (Fermentas) was used as a marker. Sizes of marker bands in kilodaltons are indicated on the left. The prominent band closely below the 40 kDa marker band corresponds to spDnmt2. Samples containing Q34tRNA^{Asp} show an additional double band below 55 kDa

compared to G34tRNA^{Asp} containing samples. These correspond to the two subunits of the TGT heterodimer that was used to enzymatically incorporate queuine into the tRNA.

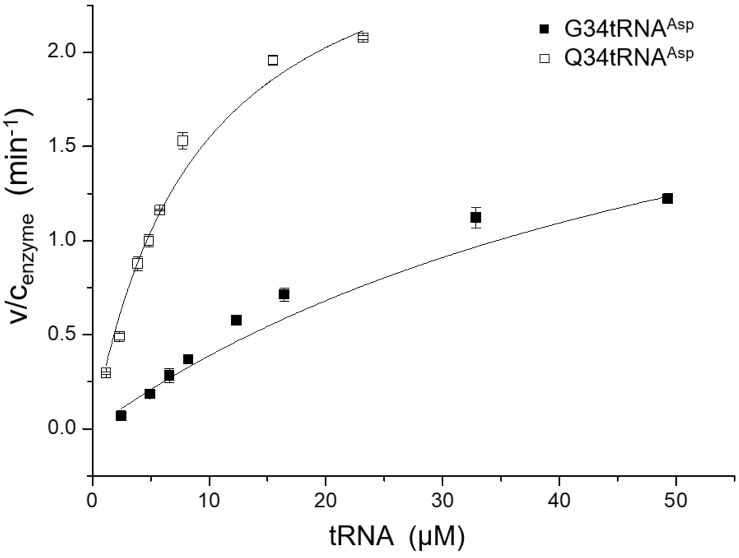

	V_{max}(min^{-1})	K_m (µM)
G34tRNA^{Asp}	2.76 ± 0.45	61.16 ± 14.55
Q34tRNA^{Asp}	2.94 ± 0.13	8.98 ± 0.74

Supplementary Figure S6. Michealis-Menten fit of Dnmt2 Methyltransferase activity data using unmodified tRNA^{Asp} (G34tRNA^{Asp}) and queuine harboring tRNA^{Asp} (Q34tRNA^{Asp}) as substrates.

Activity is plotted as substrate conversion per enzyme concentration in min^{-1}. Measurements were performed as independent triplicates with increasing substrate concentrations. Errors are presented as standard deviation. Data points were fitted by employing the Hill equation with n fixed to 1.
